# Synchronously Intracavity-Pumped Picosecond Optical Parametric Oscillators for Sensors

**DOI:** 10.3390/s22093200

**Published:** 2022-04-21

**Authors:** Alena Zavadilová, Václav Kubeček, David Vyhlídal

**Affiliations:** Faculty of Nuclear Sciences and Physical Engineering, Czech Technical University in Prague, 11519 Prague, Czech Republic; vaclav.kubecek@fjfi.cvut.cz (V.K.); david.vyhlidal@fjfi.cvut.cz (D.V.)

**Keywords:** parametric oscillator, synchronous pumping, mode-locked Nd:YVO_4_ laser, intracavity phase interferometry, subharmonic frequency generation

## Abstract

The research and development of laser systems for intracavity phase interferometry is described. These systems are based on an intracavity synchronously pumped optical parametric oscillator (OPO), enabling the generation of two trains of picosecond pulses inside a single cavity. In such a configuration, it is possible to measure the beat note frequency between two pulses and to very precisely determine the phase difference between them. The pump source is a diode-pumped passively mode-locked Nd:YVO${}_{4}$ laser. A periodically poled magnesium-doped lithium niobate crystal is used as the optical parametric oscillator crystal coupling the pump and the signal cavities. We designed a synchronously pumped OPO in a linear and ring cavity configuration allowing generation in a dual-pulse regime. By a mutual detuning of both cavity lengths, the quasi-synchronous regime of pumping was achieved and high harmonics of repetition rate frequencies were generated. Such a system can be useful for applications such as pump-probe spectroscopy or for testing telecommunication systems. We also realized the subharmonic OPO cavity as a source of two independent trains of picosecond pulses suitable for intracavity phase interferometry; we also measured the beat note signal.

## 1. Introduction

### 1.1. Background on Intracavity Phase Interferometry

A review of the research on intracavity phase interferometry (IPI) performed by our group over the past decade is presented. This work complements a report [1] by—to our knowledge—the only other group active in this field. In IPI, the physical quantity to be measured is converted into a phase difference Δφ between two pulses sharing one laser cavity. Two pulses circulating in an active cavity give rise to two output pulse trains, or, in frequency, to two frequency combs. The resonance condition of that same cavity converts that phase difference into a frequency difference between two combs that are generated. The difference in relative phase between two circulating pulses in a linear cavity can be caused by, e.g., the electro-optic effect [2], acceleration, nano-displacements, etc., or by another effect, such as air currents [3] or nonlinear index of refraction [4]. Examples of effects that change the relative phase between the two circulating pulses in a ring cavity are air currents (Fresnel drag) [3] and rotation (Sagnac effect) [5]. In contrast to the conventional phase difference determination methods based on amplitude measurements on interference fringes, in IPI, the phase difference Δφ changes the cavity resonance condition, causing a frequency difference between counter-propagating pulses that can be measured with higher accuracy than an amplitude measurement. The method is the optical analog of the frequency-modulated (FM) radio, which is much less sensitive to amplitude noise than amplitude-modulated (AM) radio. As in traditional radio reception, the signal is a modulation of a carrier, which is, in IPI, at an optical frequency. The extraction of the frequency difference proportional to the phase to be measured, takes place in a simple quadratic optical detector where the two beams issued from the laser are made to interfere. The so-called beat note that is recorded is at the frequency

(1) $$\Delta \nu =\Delta \varphi /(2\pi \tau_{RT})$$

where $\tau_{RT}$ is the cavity round-trip time. A remarkable property of IPI is that the beat note bandwidth—which determines the accuracy of the measurement—is orders of magnitude smaller than the bandwidth of each of the frequency combs that are made to interfere. The beat note bandwidth is of the order of 1 Hz [6], while that of the individual modes of the source is in the MHz range. Because the beat note is inversely proportional to the cavity length, as shown in Equation (1), the sensitivity is improved by miniaturization. The dynamic range also improves with reduced linear dimensions, since the highest measurable beat note is half the mode spacing of the laser. An exception to the scale factor being inversely proportional to the size is the application of IPI to rotation sensing. The optical gyroscope is an IPI measurement of the Sagnac phase shift, proportional to the square of the linear dimension. In the case of an active laser optical gyroscope, the interferometer is a laser with a ring cavity.

### 1.2. Gyroscopes as a Particular Case of IPI

All active laser gyroscopes that are currently in commercial use are based on continuous gas lasers with inhomogeneous gain line broadening (usually He-Ne). If the line is homogeneously broadened, mutual gain saturation will result in unidirectional operation. At small rotation rates, the beat note response may vanish because of coupling between the counter-circulating beams caused by scattering. Back-scattering of one beam into the other causes the two beams to acquire the same frequency through mutual injection [7]. If the back-scattering on any element on which the counter-propagating pulse trains meet is strong enough, the two modes are locked. Their frequencies will then be the same, the beat note frequency will not be measurable, and the laser gyroscope will be insensitive. This area is called the dead band of the gyroscope. With cw lasers, where the beams overlap throughout the cavity, the only way to minimize the dead band is to remove all elements from inside the resonator and use expensive low-scattering optical elements.

### 1.3. Need for Ultrashort Pulses

The dead band, however, can be totally eliminated by the use of mode-locked lasers where the pulse crossing regions are in a vacuum or in optical elements that do not lead to phase coupling between the circulating pulses in the cavity [5,8]. Another factor imposing the use of ultrashort pulses for IPI is mutual gain saturation. A counter-propagating beam will contribute two times more to gain saturation of a single beam in an homogeneously broadened medium, resulting in the elimination of the weaker beam in the laser cavity. With ultrashort pulses, mutual competition leading to a one-way circulation can be completely eliminated by ensuring that the pulses do not overlap in the gain medium.

### 1.4. Various Implementations

These points were confirmed in experiments conducted using dye or Ti:Sapphire lasers [3,5]; however, these lasers are bulky and most applications require compact devices. For application to sensors, a more compact Nd:Vanadate laser pumped by a laser diode and mode locked by a quantum-well (QW) saturable absorber in reflection [9] or in transmission setup [10] has been attempted. Because this laser uses a fixed saturable absorber at the pulse-crossing region, it has a non-zero dead band, which requires transverse motion [8] or electro-optic dithering [11] to be eliminated. One would expect to achieve better wall-plug efficiency and compactness with systems based on a mode-locked fiber laser. Gyroscopic response has been demonstrated with passively mode-locked fiber lasers [12,13]. So far, fiber lasers have not matched discrete component lasers in terms of the beat note bandwidth, stability, and absence of the dead band. Creating two combs of the same mode spacing is challenging with fiber lasers, because of the strong power dependence of the group velocity in the gain fiber [14].

The total absence of the dead band is realized with optical parametric oscillators (OPO) synchronously pumped by a mode-locked laser [15]. In such a system, parametric gain exists only when the pump pulse is present. The life time of the parametric gain equals the length of the pump pulse, and therefore, there is no interaction between the pulses in the amplifying media. Because the optical parametric oscillator is seeded from both sides, the crossing point of counter-propagating beams is controlled externally by the time of arrival of the pump pulses and can be easily determined in advance so that these locations are in the air. In the realization of such a two-pulse OPO, it is essential to have the pump areas perfectly co-located for both directions.

This setup is applicable in sensor applications where the physical quantity to be measured is converted into a phase difference between the two counter-propagating pulses in the cavity [1,16]. The devices that allow us to generate two independent trains of broadly tunable picosecond or femtosecond pulses are synchronously pumped OPO [17,18]. The first demonstration of such device for IPI was presented by Meng et al. [4]. The system was based on the linear ring OPO cavity externally pumped by a femtosecond Ti:Sapphire laser. Due to the beam pointing fluctuations of the pump beam, the beat note bandwidth was quite large (≈10 Hz).

### 1.5. Intracavity Pumped OPO

The instability created by small beam pointing fluctuations of the pump laser can be prevented when the pump beam is part of the same spatial cavity mode for both directions. This was accomplished by inserting a nonlinear crystal in the resonator of the pump laser [15] and thus was realized the intracavity-pumped synchronously pumped OPO. Such systems, based on Ti:sapphire lasers, require high-power pumping, which decreases the wall-plug efficiency and increases the laser system initial and operating costs. Both trains generated by the parametric cavity have, by design, exactly the same repetition frequency. As the repetition rate of the pump drifts or fluctuates, the OPO signal will adjust its wavelength to track the pump rate through the cavity dispersion. This variation in wavelength can be used to maintain the pump and OPO cavities equal.

In contrast to previous works, when the Ti:sapphire laser was used as a pumping source, we decided to use a neodymium mode-locked laser. The Nd:YVO${}_{4}$ belongs to the well-established laser materials offering excellent optical and mechanical quality together with the high gain [19,20]. The advantage of such a laser is in the ability to pump the active media directly by a laser diode, while Ti:sapphire lasers have a low efficiency and expensive sources of pumping radiation (second harmonic frequency from diode-pumped neodymium laser). A direct diode pump simplifies the whole system and reduces its cost.

As a gain medium for the parametric oscillator, a periodically poled magnesium-doped lithium niobate crystal (MgO:PPLN) is chosen. Quasi-phase matching guaranteed by periodic poling of the PPLN crystal provides fulfillment of the phase-matching condition, which makes the OPO design simpler and less sensitive to precise setting and reduces the lasing threshold. To eliminate any potential dead band, the PPLN crystal is placed inside the pump laser cavity. In the PPLN crystal, on each passage, one signal pulse is generated or amplified by a pump pulse twice per round trip. This provides the same path for the signal and pump pulse through the PPLN crystal and high pump intensity inside the cavity. Nevertheless, as this is quite a complicated setup, it is difficult to achieve a perfect spatial overlap of the signal and pump beam in the PPLN crystal and with a stable mode locking of the pump laser. Insufficient overlap is a problem not only because it can decrease conversion efficiency, but it is also one of the parameters that influences double-pulse operation and may be the cause of a single-pulse regime.

### 1.6. Summary

We have developed an intracavity synchronously pumped OPO, generating two independent trains of picosecond pulses [21,22,23] suitable for intracavity interferometry. IPI based on two equal and correlated combs, which we are investigating, has, in comparison with standard dual-comb spectroscopy [24,25], the advantage of higher sensitivity and narrower bandwidth [26]. First, the linear and ring OPO cavity enabling the dual-pulse regime generation is introduced. For those systems, we also realized the pulse-frequency multiplication regime. Finally, we present the beat note detection in the sub-harmonic configuration of the OPO.

## 2. Experiment

All systems presented here consist of two main parts: the quantum-well mode-locked Nd:YVO${}_{4}$ pump laser and the PPLN-based OPO. The pump laser was optimized with regard to application requirements for synchronous pumping and remained unchanged for all experiments presented here. Details of the laser systems are described in the following subsections.

### 2.1. Pump Laser for Synchronous Pumping of Optical Parametric Oscillators

**Figure 1:**
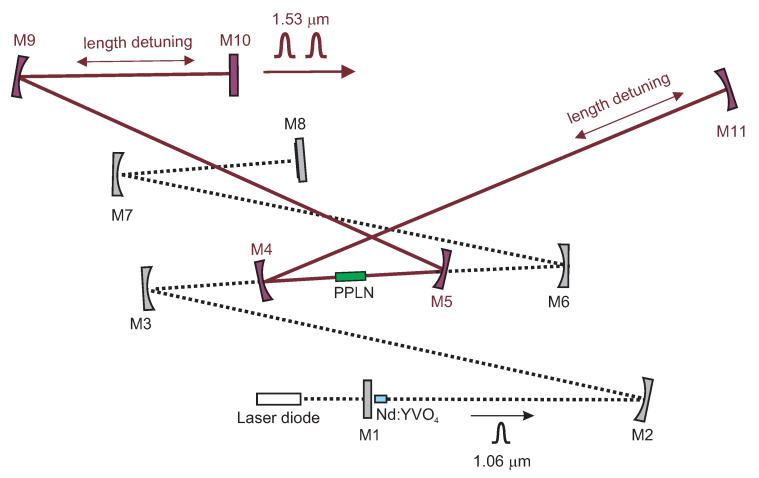
Sketch of the diode-pumped mode-locked Nd:YVO${}_{4}$ laser with a linear intracavity OPO. M1—flat mirror, M2—spherical mirror (*r*=0.5 m), M3, M6, M7—spherical mirrors (*r*=0.3 m), all highly reflective at 1.06 μm, LD—fiber-coupled laser diode, M8—QW saturable absorber combined with mirror, PPLN crystal, M4, M5—spherical mirrors (HR @ 1.53 μm, HT @ 1.06 μm, *r*=0.15 m), M10—flat output mirror of OPO resonator (T=11% @ 1.53 μm), M9, M11—spherical mirror (HR @ 1.53 μm, *r*=5 m).

The pump laser cavity is designed so that the resonator mode diameter at the location of the active laser crystal matches the laser diode beam, the beam radius at the nonlinear crystal is as small as possible, and the highest possible radiation intensity is achieved at the QW. The active medium of the pump laser is anti-reflection coated 0.5 at.% Nd:YVO${}_{4}$ crystal with a square cross-section ($5\times 3\times 3\;{\mathrm{mm}}^{3}$) placed in a water-cooled copper holder. The crystal is pumped by a 20 W 808 nm laser diode (HLU_20F400-808 LIMO GmbH, Dortmund, Germany now part of Focuslight Group, Xi’an, China) through an optical fiber of 400 μm diameter and a numerical aperture of 0.22. The folded Nd:YVO${}_{4}$ laser resonator comprises the following mirrors (see Figure 1): flat pumping mirror M1 (T>95% @ 808 nm, R>99.5% @ 1064 nm), concave folding mirror M2 (*r* = 500 mm), pair of concave mirrors M3 and M6 (*r* = 300 mm) focusing the radiation inside the PPLN crystal, and concave mirror M7 (*r* = 300 mm) focusing the radiation on the M8 of the end mirror with a saturable absorber (SAM-1064-1-25.0s-10ps, BATOP GmbH, Jena, Germany). All the mirrors are highly reflective (HR) @ 1064 nm. The cavity is designed to be insensitive to the thermal lens effect in the active medium for a wide range of pumping power. The design is described in detail in references [27,28]. The OPO part is detailed in the following section.

### 2.2. Optical Parametric Oscillator with Linear Cavity

The experimental setup is shown in Figure 1. As a nonlinear medium for the OPO, a periodically poled MgO-doped LiNbO${}_{3}$ crystal (PPLN) is used. We subsequently tested two types of crystals. The first crystal had dimensions of 20×3×1 ${\mathrm{mm}}^{3}$ with a domain period of 30.3 μm. This poling period corresponds to the OPO signal wavelength of 1526 nm @ 1064 nm pump at 298 K. The crystal has Brewster angle cut faces, which induced a strong astigmatism. This aberration had to be compensated for by adjusting the spherical mirrors M4 and M5 (*r* = 150 mm, HR @ 1.5 μm). These mirrors were forming, at the same time, the focusing Newton-type telescope providing higher laser intensity in the PPLN crystal with no influence on the OPO cavity mode.

The second tested crystal had dimensions of 20×10×1 ${\mathrm{mm}}^{3}$ with 5 domain periods from 29.52 to 31.59 μm, which corresponded to the signal wavelengths from 1480 to 1620 nm @ 1064 nm pumping at 298 K (MOPO1-1.0-2 Covesion Ltd., Southampton, UK).

The results obtained using those crystals were similar; however, the configuration with the Brewster angle cut crystal was harder to set up because of the more complicated alignment (different paths for different wavelengths). The generated wavelength of the OPO was tuned by changing the PPLN crystal’s temperature. The crystal’s temperature was controlled using a special copper mount and could be set in the range from 25 to 80 ${}^{{}^{\circ}}$C.

### 2.3. Optical Parametric Oscillator with Ring Cavity

**Figure 2:**
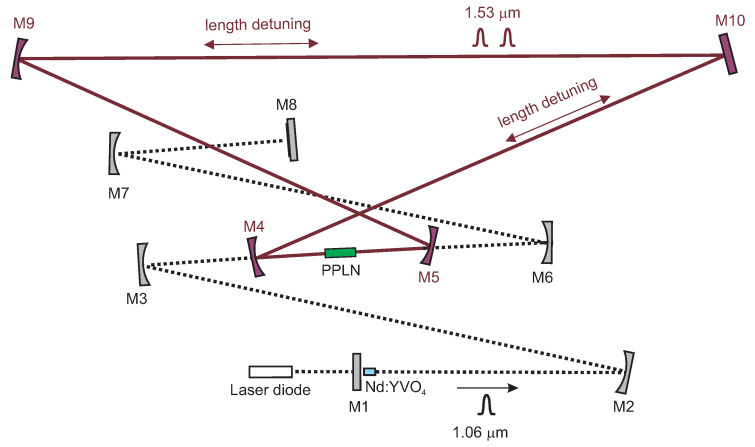
Diagram of diode-pumped mode-locked Nd:YVO${}_{4}$ laser with an intracavity ring OPO. M1—flat mirror, M2—concave mirror (*r*=0.5 m), M3, M6, M7—concave mirrors (*r*=0.3 m), all HR @ 1.06 μm, M8—semiconductor saturable absorber mirror, PPLN crystal, M4, M5—concave mirrors (HR @ 1.53 μm, HT @ 1.06 μm, *r*=0.15 m), M9—concave mirror (*r*=3 m, HT @ 1.53 μm), M10—flat mirror (T=11 % @ 1.53 μm).

We continued with the knowledge obtained during the implementation of the linear OPO cavity and realized the ring OPO cavity. This optical parametric resonator was designed as a ring, slightly focusing stable cavity, and included a curved long-radius mirror M9 (*r* = 3 m, HR @ 1.5 μm) and an output coupler M10, (T=11% @ 1.53 μm). The setup is presented in Figure 2.

## 3. Results

The results concerning the dual-pulse regime and overall system stability in linear and ring cavity OPO pulse-multiplication regime achieved for those setups and beat note detection in subharmonic configuration of linear cavity are listed in the following sections.

### 3.1. Laser Performance

The threshold pump power of the Nd:YVO${}_{4}$ laser was 2 W; the stable continuous mode-locked operation was attained at a pump power of 7 W. All measurements were performed at an operating point corresponding to the pump power of 10 W. The output power was measured by a power meter FieldMate (Coherent, Inc., Santa Clara, CA, USA) with a detector Molectron PM10 (Coherent, Inc., Santa Clara, CA, wavelength range 190 nm–11 μm, power range 0.01–10 W). The OPO signal beam power was measured to be from 0.4 to 4 mW depending on the laser settings and stability (measured behind the mirror M11). The output power was measured by optical power meter Thorlabs PM30 (Thorlabs Inc., Newton, NJ, USA) with GE detector S122B (Thorlabs Inc., Newton, NJ, USA, wavelength range 700 nm–1.8 μm, power range 35 nW–35 mW).

In the current setup, we observed several wavelengths in the output spectrum of the overall laser system. These were present: the second harmonic of the pump (532 nm), the sum of the pump and signal (628 nm), the pump (1064 nm), the signal (1534 nm), and the second harmonic of the signal wavelength (767 nm). It was possible to tune the signal wavelength by changing the PPLN crystal temperature. The sum of the pump and the signal was used as a visible sign of parametric generation and the quality of the beam.

Pump pulse duration was measured by a laboratory-designed noncollinear second-harmonic-generation-based autocorrelator and was determined to be 15 ps (FWHM). For the long term stability measurements, an EOT photodetector ET-3000 (Coherent, Inc., Santa Clara CA, USA, rise time <175 ps) was used. The pulse-repetition rate was measured to be ∼82.6 MHz.

### 3.2. Intracavity Synchronously Pumped OPO with Linear Cavity

Intracavity-pumped OPO seemed to be a routine in the early 1990s with mode-locked dye lasers [29]. Intracavity pumping is considerably more challenging with long relaxation time gain media—such as Ti:sapphire and YVO${}_{4}$—which have a tendency to Q-switch; a tendency exacerbated by coupling with the OPO cavity. Stabilization methods either by nonlinear losses [22] or by complex dispersion manipulation [30] have been proposed; however, to our knowledge, these have never been implemented. We were nevertheless able to find a range of parameters leading to stable operation.

**Figure 3:**
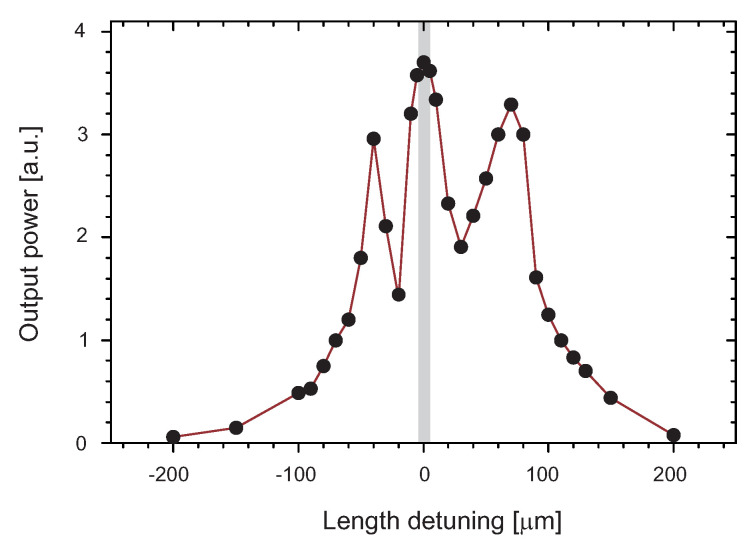
Dependence of the OPO signal power measured behind M6 (R=89% @ 1.5 μm) on the length detuning of cavities. The area of stable mode locking is highlighted in gray.

**Figure 4:**
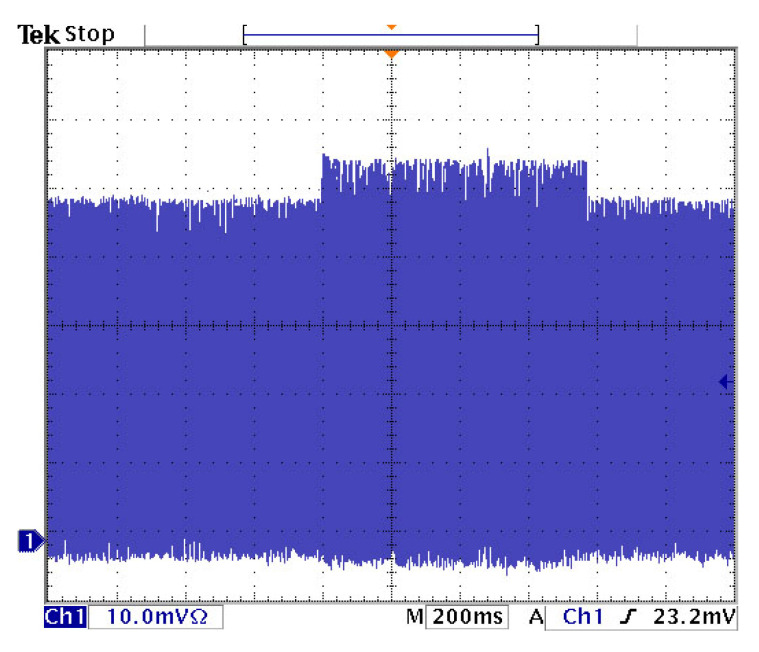
Oscillogram of pump laser pulse when OPO is in cw mode-locked regime (lower level) and when OPO generation is blocked (upper level).

To achieve the OPO generation in such a complex setup, the sensitivity of the generation of continuous mode-locking depending on mutual length detuning of both cavities was tested. It was found that the range of stability was only ±5 μm. When the detuning exceeded ±200 μm parametric generation was no longer present. The dependence of the OPO power on the length detuning of cavities measured behind the mirror M6 is shown in Figure 3. It was also possible to observe an increase in the intensity of the pump laser pulses when one of the mirrors of the optical parametric oscillator was blocked and the parametric generation was disabled, see Figure 4. From the change in intensity, it was possible to deduce the amount of energy converted into the signal wave.

**Figure 5:**
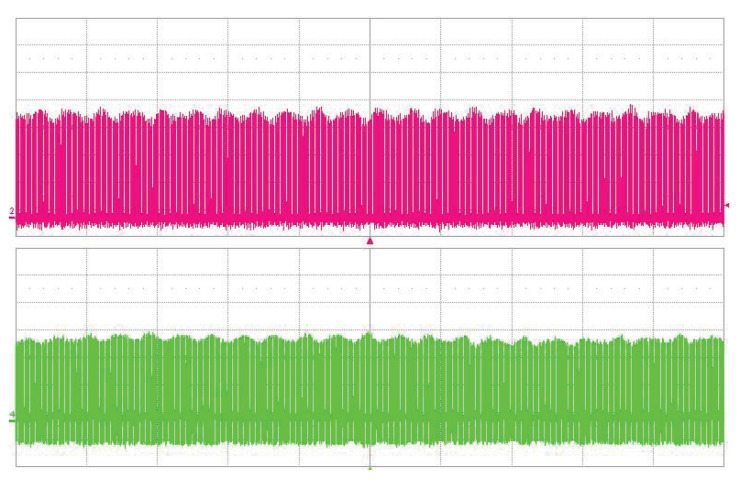
Oscillogram of dual-pulse regime. 1.06 μm pump (upper trace), PPLN OPO radiation at 1.53 μm (lower trace). Time scale is 500 ns/div.

**Figure 6:**
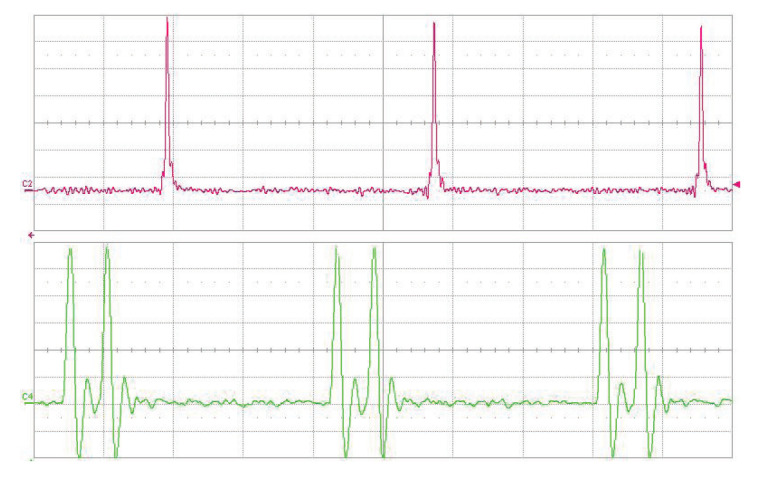
Oscillograms of dual-pulse regime, 1.06 μm pump (upper trace), PPLN OPO radiation at 1.53 μm (lower trace). Time scale is 2 ns/div.

The long-term stability of the OPO signal was measured behind the mirror M9 by EOT photodetector ET-3500 (Coherent, Inc., Santa Clara CA, USA, rise time <25 ps) connected to the LeCroy oscilloscope SDA9000 (Teledyne LeCroy, Inc., Chestnut Ridge NY, USA, analog bandwidth 9 GHz, sampling rate 40 GS/s). A weak modulation of the pulse train can be seen in both traces— Figure 5. With careful alignment, both single and double-pulse regimes of the OPO were obtained. In the double-pulse regime, for each pump pulse, a pair of signal wave pulses were generated, as can be seen in Figure 6. This confirms the two-way generation of parametric radiation. The signal pulses were generated when the pump pulse went back and forth through the PPLN crystal after the reflection from the saturable absorber (M8). The distance between the two pulses is given by twice the difference in optical distances between mirror M8 and PPLN and PPLN and mirror M11. In this case, the observed pulse delay is 1.5 ns.

**Figure 7:**
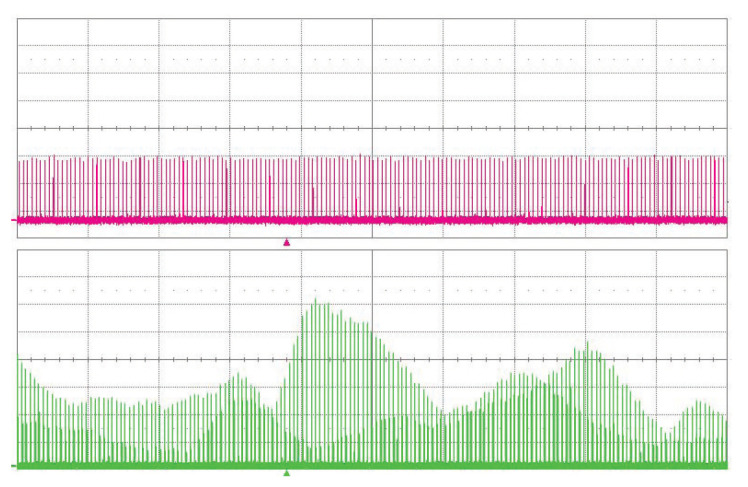
Oscillograms of dual-pulse regime, competition between both trains, 1.06 μm pump (upper trace), PPLN OPO radiation at 1.53 μm (lower trace). Time scale is 200 ns/div.

The stable double-pulse regime was hard to maintain and was very sensitive and could be easily distorted. At longer time-scale the competition between the two pulses in the parametric cavity occurred easily. A typical situation can be seen in Figure 7. The energy is transferred from one pulse train to the other and vise versa. Any random air flow or mechanical disturbance can cause a preference of one OPO train and the laser switches to the single-pulse regime.

### 3.3. Synchronously Intracavity Pumped OPO with Ring Cavity

**Figure 8:**
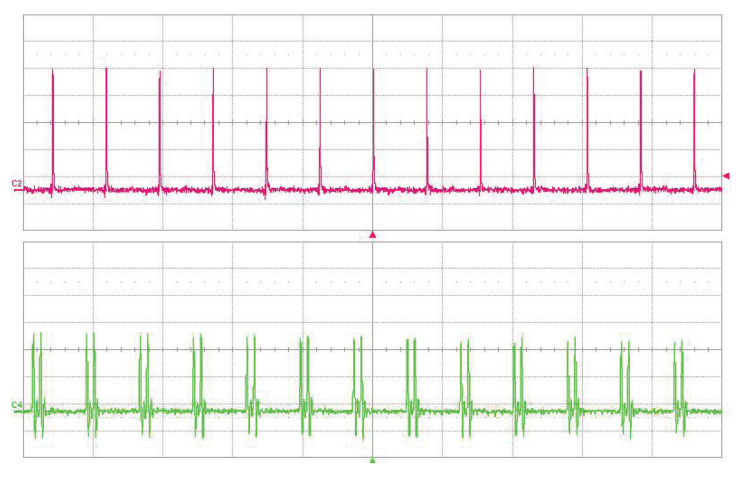
Oscillogram of dual-pulse regime. 1.06 μm pump (upper trace), PPLN OPO radiation at 1.53 μm (lower trace). Time scale 10 ns/div.

Analogous to the linear OPO cavity arrangement, the goal for a ring cavity was also to achieve a generation of double pulses circulating inside the cavity. In the case of the ring cavity, the OPO should be twice the length of the pump cavity. We observed that the possible OPO cavity length detuning (compared to the length of the pump laser) in the vicinity of $2L_{pump}$ enabling parametric oscillations has to be smaller than 100 μm. In this configuration, we also achieved the dual-pulse OPO. The signal was measured behind mirror M10. In the direction from mirror M9, the signal corresponds to the pulses propagating in the counterclockwise direction; from mirror M5, the signal corresponds to the clockwise direction. In the double-pulse regime, for each pump pulse, a pair of signal wave pulses were generated as can be seen in Figure 8. This confirms two-way generation of parametric radiation.

**Figure 9:**
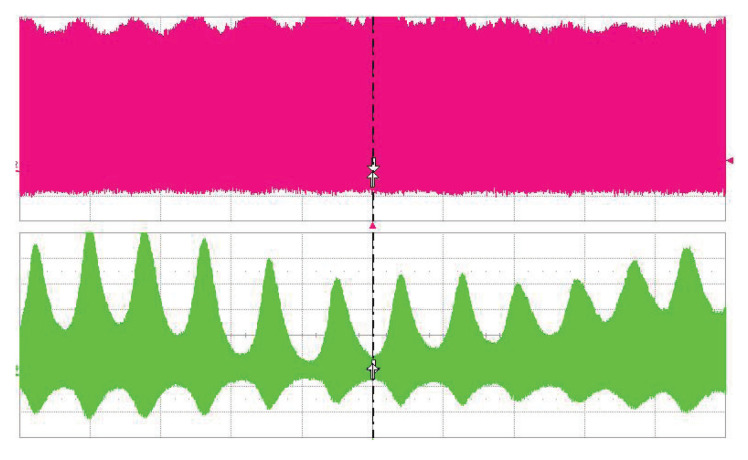
Oscillogram—unstable dual-pulse regime. 1.06 μm pump (upper trace), PPLN OPO radiation at 1.53 μm (lower trace). Time scale 1 μs/div.

This setup was even more tricky to adjust. The OPO resonator arm length detuning also changed the angle of incidence on the mirrors and the adjusting caused further changes of the length. System operation was very sensitive to any perturbations; we observed fluctuations in signal and pump pulse amplitude as seen in Figure 9.

### 3.4. Repetition Rate Frequency Multiplication in Synchronously Intracavity Pumped Linear OPO

In the cavity of synchronously intracavity-pumped OPO, it was possible to achieve the regime of pulse multiplication. This so-called quasi-synchronous pumping enabled not only the generation of the OPO pulse with a repetition rate corresponding to the pump pulse repetition rate but also its higher harmonics.

**Figure 10:**
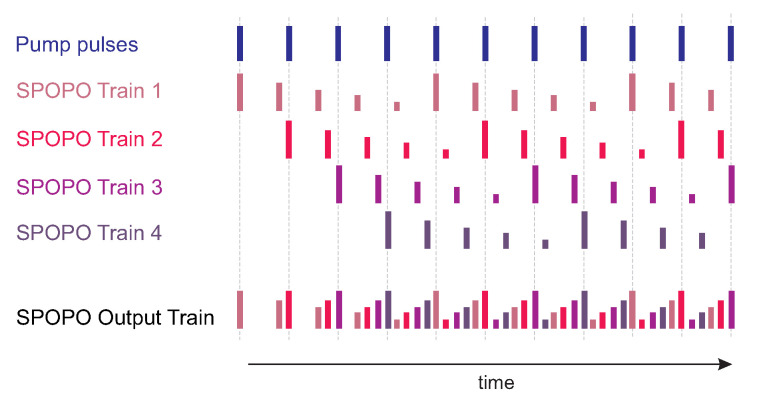
The principle of the pulse multiplication, factor of multiplication n=5.

The principle is as follows: In the synchronously pumped OPO, the length of the linear OPO cavity $L_{opo}$ should be equal to the length of the pump cavity $L_{pump}$. With equal lengths, the repetition rate of both lasers is equal and each pump pulse corresponds to the one in the OPO. When the OPO cavity is shorter and the difference between the mutual resonators lengths $\delta =L_{pump}/n$ where *n* is an integer, the synchronous pumping occurs after *n* round trips of the OPO cavity that corresponds to n−1 round trips of the pump resonator. The repetition rate of the OPO will be *n*-times higher than that of the pump [31]. The principle of the pulse multiplication is explained graphically in Figure 10. Because there is no gain for the signal pulse inside the PPLN crystal without simultaneous co-propagation with the pump pulse, a successive gradual decrease in the OPO pulse amplitude will occur until the next pump pulse arrives and provides energy. The increase in signal pulse amplitudes between two pumping pulses observed on oscillograms was given by the fact that the length of the OPO was shorter than the pump cavity, and the pulses remained longer in the OPO cavity after the amplification.

**Figure 11:**
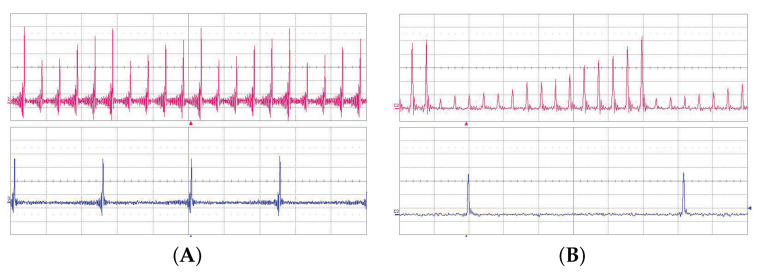
Oscillograms of the signal linear cavity OPO radiation at 1.53 μm (upper trace) and 1.06 μm pump (lower trace). (**A**) Pulse multiplication factor n=5, time scale 5 ns/div. (**B**) Pulse multiplication factor n=15, time scale 2 ns/div.

**Figure 12:**
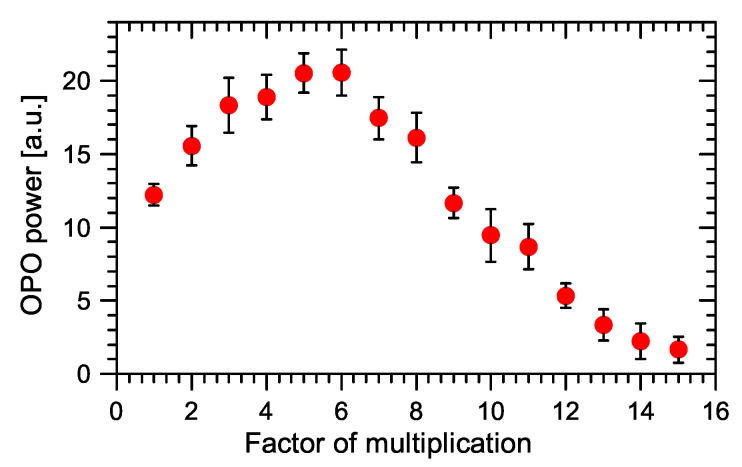
Dependence of OPO output power behind M10 on multiplication factor in the linear OPO cavity.

**Figure 13:**
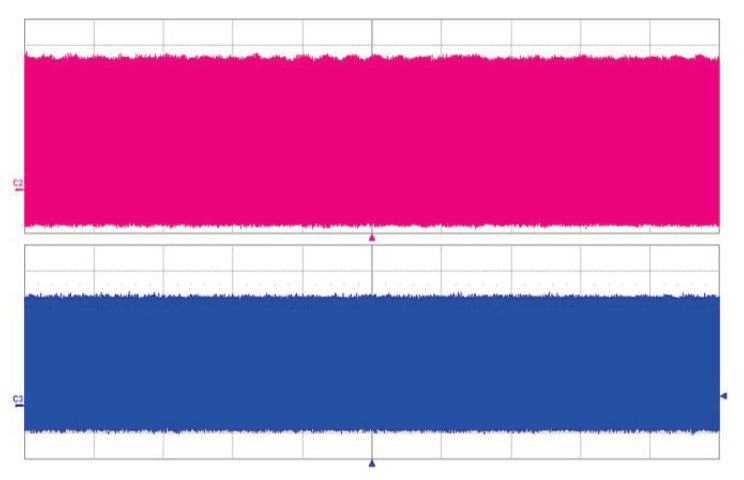
Oscillogram of the signal OPO radiation at 1.53 μm (upper trace) and 1.06 μm pump (lower trace)—long-time stability, factor of multiplication n=13. Time scale 5 μs/div.

The value of the cavity lifetime limited the possible multiplication factor *n*. We have successfully proved the pump multiplication of our linear OPO for factor *n* from 1 to 15. The basic setup of the cavity is shown in Figure 1. The examples of oscillograms of the signal OPO radiation for pulse multiplication factor n=5 and n=15 are in Figure 11. The dependence of the OPO output power measured behind mirror M10 on the factor of multiplication *n* is in Figure 12. The output power variations with *n* can be explained by the change of effective OPO outcoupling with the increasing *n*. From this, it follows that the increase of *n* decreases the amplitude of the signal pulse by interacting with the pump pulse inside the nonlinear medium. This caused a smaller depletion of pump pulse amplitude, which reduced the nonlinear coupling between pump and OPO cavities, leading to better stability of the laser system. A similar effect should be obtained by changing the OPO output coupler losses, but changing the *n* is more flexible. An example of measured long-time stability is presented in Figure 13 for (n=13). In this case, only small fluctuations of OPO signal were present and the modulation of the pump pulse amplitude was negligible.

The quasi-synchronous pumping of the OPO, based on the precise mutual length change of the pump and the OPO cavity length has been already demonstrated. Using the Ti:Sapphire femtosecond laser extra-cavity-pumped linear OPO 1 GHz pulses up to the 14th harmonic were generated [32]. We demonstrated the generation of 1.2 GHz OPO repetition frequency corresponding to 15th harmonic of pump pulse in linear intracavity-pumped OPO.

### 3.5. Repetition Rate Frequency Multiplication in Synchronously Intracavity-Pumped Ring OPO

The frequency multiplication principle in synchronously pumped ring OPO is analogous to that described in the previous section. The mutual cavities length difference (shortening in our case) was $\delta =2L_{pump}/n$ where *n* is an integer, the synchronous pumping occurred after *n* round trips of the OPO cavity corresponding to n−1 round trips of the pump cavity and the repetition rate of the OPO was *n*-times higher than the pump repetition rate [31,33,34].

**Figure 14:**
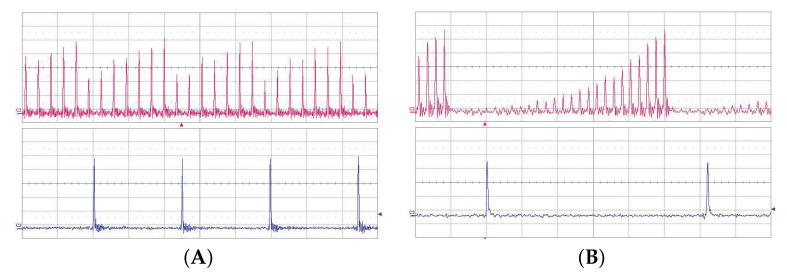
Oscillogram of the signal ring cavity OPO radiation at 1.53 μm (upper trace) and 1.06 μm pump (lower trace). (**A**) Pulse multiplication factor n=7, time scale 5 ns/div. (**B**) Pulse multiplication factor n=27, time scale 2 ns/div.

We have successfully achieved OPO repetition rate frequency multiplication for factor *n* from 2 to 28. Oscillograms of the signal OPO trains for pulse multiplication factor 7 and 27 are shown in Figure 14. High-repetition rate (2.27 GHz) signal pulse train was achieved for the multiplication factor n=28.

**Figure 15:**
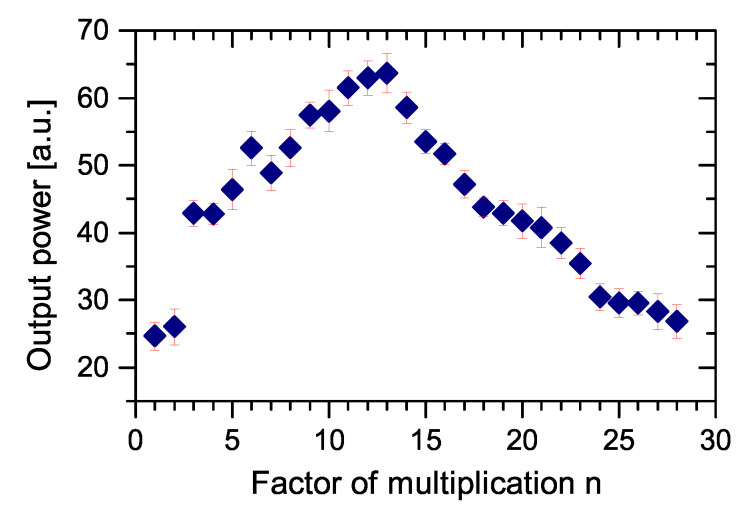
Dependence of OPO output power behind M10 on multiplication factor in the ring OPO.

**Figure 16:**
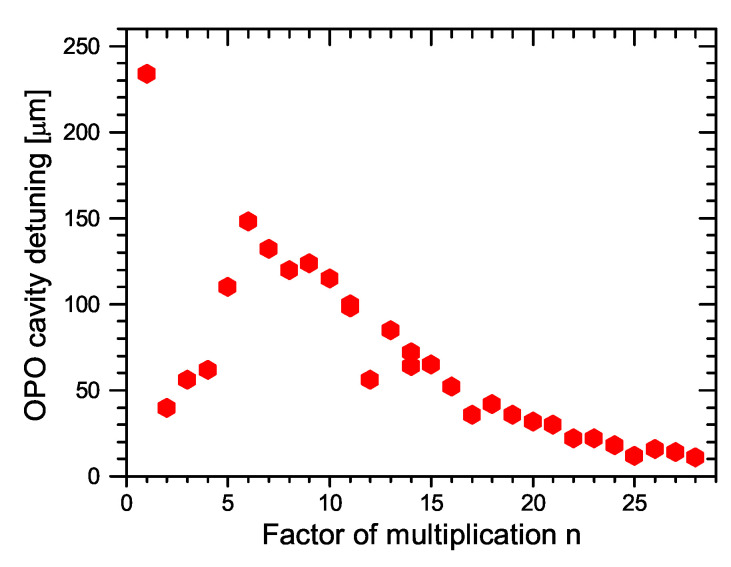
Dependence of resonator length detuning enabling the generation on the factor of multiplication *n* in the ring OPO.

Dependence of the OPO output power behind mirror M10 on the factor of multiplication *n* is shown in Figure 15 and the dependence of OPO resonator length detuning still enabling the OPO generation on a factor of multiplication *n* is presented in Figure 16. It can be seen that, for higher *n*, the tolerance is smaller and the setting must be more accurate. In this case, only small fluctuations of the OPO signal were present and the modulation of the pump pulse amplitude was negligible. The OPO signal behind mirror M10 was detected simultaneously in both directions, confirming the bidirectional operation of the OPO. The possible detuning allowing parametric generation was significantly shorter than for n=1. This could be caused by higher effective losses of the OPO cavity.

We can conclude that we have successfully demonstrated the multiplication of the pump repetition rate inside the ring synchronously intracavity-pumped OPO induced by precise mutual change of the pump and OPO cavity length and this system could be used as a widely tunable high repetition rate pulse generator. We have also observed that, by appropriate selection of the multiplication factor, the coupling between both cavities could be controlled, leading to an increase in overall laser system stability.

### 3.6. Quasi-Synchronous Pumping and the Beat Note Detection

In Section 3.4, dual-frequency combs generation was presented but the intracavity OPO was very sensitive to any disturbance and has a tendency to be unstable. Competitive OPO frequency combs may occur due to the system asymmetry [35]. The first laser comb is pumped by the pulse heading from the gain medium; the second comb in the opposite direction is pumped by the reflected and drained pump pulse. Another problem was to obtain the identical path for both laser combs in the PPLN crystal. Several techniques of stabilization were proposed, but this problem was not still fully resolved.

**Figure 17:**
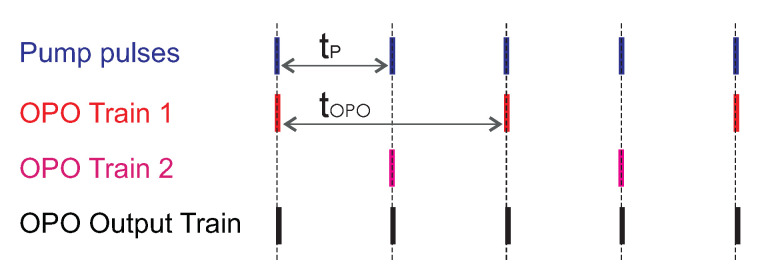
Scheme of subharmonic OPO generation, OPO cavity length is the double of the pump cavity length. $t_{p}$ is pump round-trip time, $t_{OPO}$ is OPO round-trip time.

The method to overcome some of these limitations, at least for the linear OPO configuration, was demonstrated for extracavity pumping by A. Velten [36]. In this article, quasi-synchronous pumping of the OPO by an external Ti:Sapphire laser is described; the repetition rate of the pumping pulses was twice the repetition rate of the OPO cavity. We adopted this technique for the intracavity quasi-synchronously pumped linear OPO. The system is based on the PPLN parametric oscillator and is pumped by the diode-pumped Nd:YVO${}_{4}$ laser. In principle, it is a divide-by-2 subharmonic OPO. The graphical representation of divide-by-2 OPO trains is shown in Figure 17.

The arrangement was based on the previous linear setup (see Figure 1). In this case, the optical length of the pump cavity was set to be half of the OPO cavity length. The OPO cavity mirrors were adjusted to ensure the parametric oscillation only for pump pulses coming from the direction of the Nd:YVO${}_{4}$ crystal. This arrangement ensures stable, long-term OPO and pump laser generation without any fluctuations. In this setup, the OPO generates signal pulses with the same repetition frequency as the pump laser, but the signal consists of two completely independent pulse trains. The repetition rate of each of the two pulse trains was the half of the pump, in this particular case it was 41.1 MHz. The relative phase of these two combs was not constant because each train was built up independently from random noise [32,33,37], so such pair of independent combs is suitable for intracavity interferometry.

**Figure 18:**
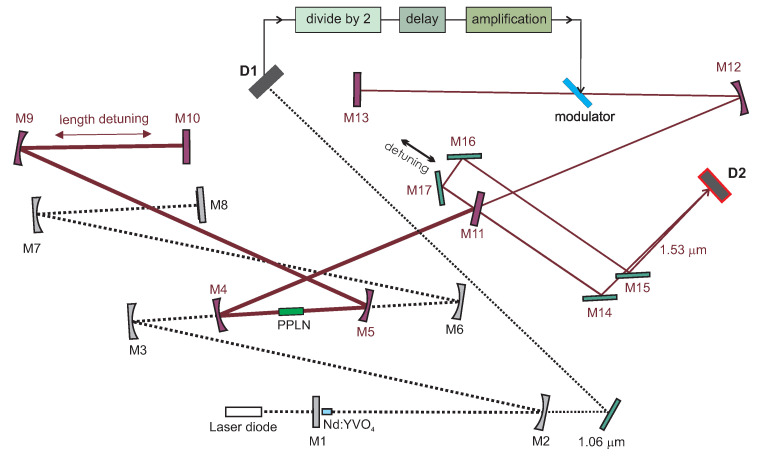
Diagram of diode-pumped modelocked Nd:YVO${}_{4}$ laser with quasi-synchronous-pumped intracavity OPO. M1—flat mirror, M2—concave mirror (*r*=0.5 m), M3, M6, M7—concave mirrors (*r* = 0.3 m), all HR @ 1.06 μm, M8—semiconductor saturable absorber mirror (QW), PPLN crystal, M4, M5—concave mirrors (HR @ 1.53 μm, HT @ 1.06 μm, *r*=0.15 m), M9, M12—concave mirror (*r* = 3 m, HR @ 1.53 μm), M10—flat mirror (T=5 % @ 1.53 μm), M12—flat mirror (T=50% @ 1.53 μm), M13, M14, M16, M17—flat mirror, HR @ 1.53 μm.

To prove the applicability and to measure the capabilities of the presented intracavity quasi-synchronously pumped OPO, we modulated the phase of one of the two pulse trains and monitored the beat note frequency between them. For this purpose, a laboratory-built electro-optic modulator (EOM) based on an LiNbO${}_{3}$ thin plate (thickness 1.5 mm, width 3.3 mm) was placed under Brewster angle inside the OPO resonator. It was placed close to the OPO cavity mirror M13. The setup is shown in Figure 18. The laser beam polarization vector lays in the direction of the EOM optical axis and was perpendicular to the laser propagation direction. The modulated electric field was applied in the direction of the EOM optical axis. The EOM was driven by 50 % duty cycle rectangular pulse signal with the frequency of 41.1 MHz (1/2 of the pump pulse frequency). This signal was created by a laboratory-designed divide-by-2 circuit. The signal derived from the laser pulses was picked up by the photodiode D1 (EOT ET-3500). The amplified output from the photodiode was fed to a fast D flip-flop wired as a divide-by-2 circuit. The output signal had a variable amplitude $V_{0}\in$ (0.1–10 V) and low amplitude pulse-2-pulse ripple (less than 2 mV). This output had to be delayed so that one pulse train got through the EOM during the high-level state of the output signal while the other pulse train during the low-level state. Due to the EOM position the modulated pulse train was phase shifted twice per round-trip. A similar arrangement is used and described in [38].

The beat note measurement was performed with a slow photodiode. Both pulse trains were extracted from the OPO by a beam splitter (semitransparent mirror M11) and directed to the slow photodiode D2 (FGA21 InGaAs photodiode, Thorlabs Inc., Newton, NJ, USA, $t_{r}$ = 66 ns) where they interfered. The photodiode was connected to 1 MegΩ oscilloscope input. Mirror M11 was inserted inside the OPO cavity, close to the crossing region of the pulse trains. To obtain spatial overlap of both pulse trains, a delay line (mirrors M14 to M17) was inserted in the path of one pulse train.

**Figure 19:**
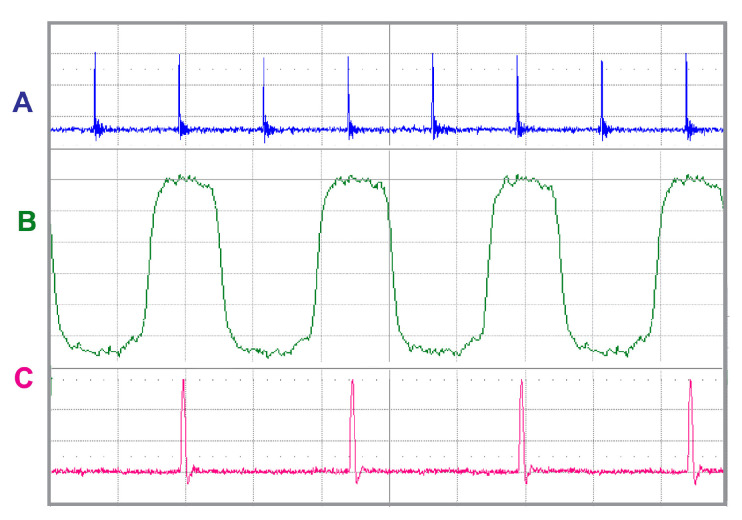
A—Train of pump laser pulses, repetition rate $f_{p}$ = 82.2 MHz. B—modulating pulses with repetition rate $f_{p}/2$, signal is derived from the pump laser pulses, C—Train of OPO pulses after the division by two and appropriate delay, repetition rate $f_{p}/2$ = 41.1 MHz.

The timing relation between both pulse trains and divide-by-2 circuit output is shown in Figure 19. The signal pulse train has the same frequency ($f_{p}$) as the pump and the OPO laser (trace A) and is used as an input to the divide-by-2 circuit whose output is a train of rectangular pulses with the frequency of $f_{p}/2$ (trace B). The resulting train of OPO pulses with half the pump frequency $f_{p}/2$ and appropriate delay is shown in trace C.

**Figure 20:**
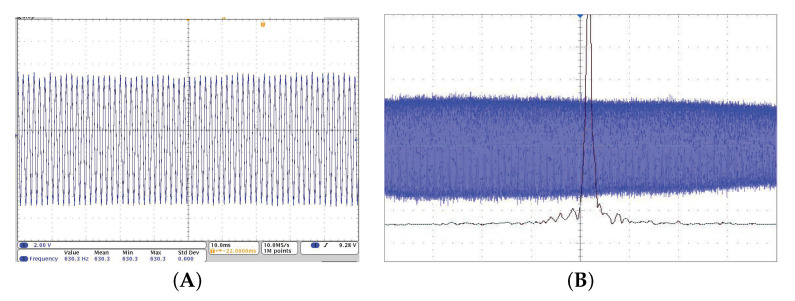
(**A**) The sample of detected beat note signal for modulation signal amplitude 275 mV. (**B**) spectrum of the beat note signal calculated using FFT from 20 s beat note record, frequency 460 Hz. The beat note bandwidth at ∼20 dB was 4.3 Hz.

**Figure 21:**
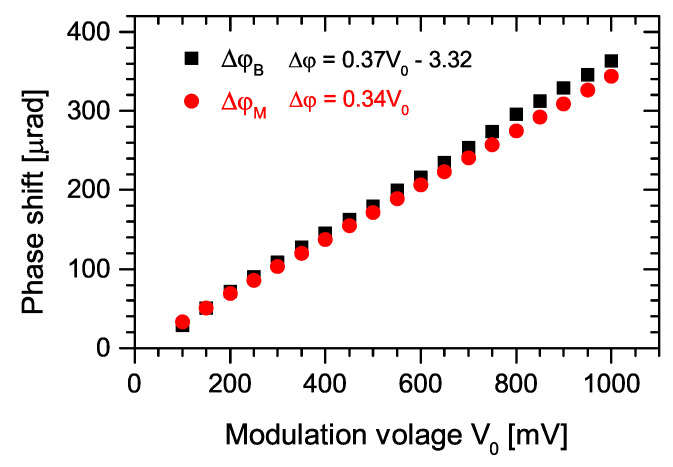
Phase shift difference as a function of applied modulator voltage: $\Delta \varphi_{B}$—phase shift difference calculated from the measured beat note frequency for given modulator voltage, $\Delta \varphi_{M}$—phase shift difference calculated from applied voltage and material parameters of lithium niobate.

Without the signal from the divide-by-2 circuit, the beat note was not present. Once this signal was applied to the EOM, the registered beat note was stable. The oscilloscope record of the measured beat note for signal amplitude 275 mV is shown in Figure 20A. The FFT was used to calculate the spectrum of the beat note signal from samples 20 s long (frequency resolution 0.05 Hz). The beat note bandwidth at ∼20 dB was 4.3 Hz. The record is shown in Figure 20B and FWHM of the beat note was 0.72 Hz. The beat note spectrum slightly fluctuated in time but its width was still limited to ∼20 Hz level and only one beat note frequency was present for long time periods. These fluctuations could be explained by divide-by-2 output amplitude instability or by some mechanical disturbances and random air flow. The dependence of the beat note frequency $f_{beat}$ on applied divide-by-2 circuit amplitude was measured and the corresponding phase shift difference $\Delta \varphi_{B}$ was calculated using following formula [16]:

(2) $$\Delta \varphi_{B}=2\pi t_{OPO}f_{beat},$$

where $t_{OPO}$ is OPO round-trip time. The result is presented in Figure 21. The lowest observed phase-shift difference between the OPO signal combs was ∼28 μrad, which corresponds to the minimum divide-by-2 circuit output amplitude of 100 mV and the beat note frequency was 182 Hz. The highest measured beat note frequency was 2320 Hz, which corresponds to the divide-by-2 circuit output amplitude of 1000 mV. In all ranges of modulation amplitudes, the FWHM of the beat note spectrum was less than 0.9 Hz, which corresponds to a phase shift measurement error of less than $1.4\times 10^{-7}$ rad.

For comparison, the theoretical phase shift difference $\Delta \varphi_{M}$ calculated from the electro-optical effect is also presented in Figure 21. When neglecting the Brewster angle of the EOM, the phase difference between the two pulse trains in EOM is related to the applied voltage $V_{0}$ by [39]:

(3) $$\Delta \varphi_{M}=\frac{V_{0}\cdot \pi \cdot r_{33}\cdot n_{e}^{3}\cdot l}{d},$$

where $n_{e}$ is the extraordinary index of refraction, $r_{33}$ electro-optic coefficient, *d* distance between electrodes, and *l* is the length of the crystal in the direction of laser beam propagation. For the calculations of $\Delta \varphi_{M}$, we used the following LiNbO${}_{3}$ material parameters: $n_{e}=2.2$ at 1.5 μm and $r_{33}=3.1\times 10^{-10}$ m/V [40]. In the ideal case, the slope of both phase shift difference ($\Delta \varphi_{B}$ and $\Delta \varphi_{M}$) dependencies on EOM modulation voltage should be identical. The observed slight difference could be caused by the nonhomogenous electric field inside the laboratory-built electrooptic modulator or inaccurately determined material parameters of lithium niobate. Nevertheless, this difference can be stated as a good agreement between the measured and the theoretical value of the phase shift difference. This arrangement can be used to measure the frequency dependence of nonlinearities.

## 4. Conclusions

We have demonstrated the achievement of two independent combs generation in a single cavity of synchronously intracavity-pumped optical parametric oscillator in linear and ring geometry of the signal cavity. The systems were based on the SESAM-modelocked, picosecond, diode-pumped Nd:YVO${}_{4}$ 1.064 μm laser and tunable 1.53 μm PPLN parametric oscillators. The influence on external conditions on system stability was also investigated.

Next, we have shown that with these systems, it is possible to realize quasi-synchronous intracavity pumping in such OPOs. This was achieved by multiplication of the pump repetition rate inside the ring intracavity synchronously pumped OPO by the precise changing of the mutual lengths of the pump and OPO cavity. In the linear configuration, the generated OPO pulse repetition rate was adjustable from 80 MHz up to 1.2 GHz. In the ring configuration, the generated pulse repetition rate was adjustable up to 2.3 GHz. These systems could be used as a widely tunable high repetition rate pulse generators or could be useful for applications such as pump-probe spectroscopy.

Another modification of this system based on the generation of subharmonic frequencies enabled us to generate two independent pulse trains in the OPO and phase shift difference associated beat note frequency caused was measured. The applicability of this system for IPI was thus successfully demonstrated. The bandwidth of the observed beat note was less than 0.9 Hz (FWHM) without any active stabilization. This arrangement can be used to measure the frequency dependence of nonlinearities.

## Data Availability

The data are contained within the article. No dataset is presented online.
